# Long noncoding RNA MAPKAPK5-AS1 promoted lipopolysaccharide-induced inflammatory damage in the myocardium by sponging microRNA-124-3p/E2F3

**DOI:** 10.1186/s10020-021-00385-1

**Published:** 2021-10-19

**Authors:** Weiwei Chen, Guangyuan Gao, Mengjie Yan, Ming Yu, Kaiyao Shi, Ping Yang

**Affiliations:** 1 Department of Cardiology, China-Japan Union Hospital of Jilin University, Changchun City, 130033 Jilin Province People’s Republic of China; 2 Jilin Provincial Key Laboratory for Genetic Diagnosis of Cardiovascular Disease, Changchun City, 130033 Jilin Province People’s Republic of China

**Keywords:** MAPKAPK5-AS1, Lipopolysaccharide, Inflammatory response, microRNA-124-3p, E2F3

## Abstract

**Background:**

Myocardial dysfunction caused by sepsis (SIMD) leads to high mortality in critically ill patients. We investigated the function and mechanism of long non-coding RNA MAPKAPK5-AS1 (lncRNA MAPKAPK-AS1) on lipopolysaccharide (LPS)-induced inflammation response in vivo and in vitro.

**Method:**

Male SD rats were utilized for in vivo experiments. Rat cardiomyocytes (H9C2) were employed for in vitro experiments. Western blotting was employed to measure protein expression, and RT-PCR was performed to measure mRNA expression of inflammation factors. TUNEL and flow cytometry were carried out to evulate cell apoptosis.

**Result:**

The results showed that the expression of MAPKAPK5-AS1 was increased, while the expression of miR-124-3p was decreased in the inflammatory damage induced by LPS in vivo and in vitro. Knockdown of MAPKAPK5-AS1 reduced LPS-induced cell apoptosis and inflammation response, while overexpression of miR-124-3p weakened the effects of MAPKAPK5-AS1 knockdown on LPS-induced cell apoptosis and inflammation response. Moreover, miR-124-3p was identified as a downstream miRNA of MAPKAPK5-AS1, and E2F3 was a target of miR-214-3p. MAPKAPK5-AS1 knockdown increased the expression of miR-124-3p, while miR-124-3p overexpression reduced the expression of MAPKAPK5-AS1. In addition, miR-124-3p was found to downregulate E2F3 expression in H9C2 cells.

**Conclusion:**

MAPKAPK5-AS1/miR-124-3p/E2F3 axis regulates LPS-related H9C2 cell apoptosis and inflammatory response.

## Introduction

Sepsis-induced myocardial dysfunction (SIMD) is one of the most serious complication in patients with sepsis (Kakihana et al. 2016; Yamaji et al. 2017). Studies have shown that inflammation, oxidative stress and cardiomyocyte apoptosis are the three key factors in the development of SIMD (Walley 2018; Yang et al. 2019). The pathogenesis of SIMD is extremely complex, with series of pathophysiological changes in the body, causing dramatic changes in hemodynamics, leading to heart failure (Lv and Wang 2016; Hashem et al. 2018). Lipopolysaccharide (LPS) is a component in the cell wall of gram-negative bacteria, which is an important initiating factor for sepsis (Wang 2016; Wu et al. 2019). LPS can induce the expression of inflammation factors in cardiomyocytes, cause cardiomyocyte hypertrophy and apoptosis and weaken the contractile function of cardiomyocytes, eventually leading to congestive heart failure (Wang et al. 2016; Tan et al. 2019). inflammatory damage in myocardium injury induced by LPS is an important factor that causes refractory hypotension and sepsis death, which has received increasing attention in research (Gichana et al. 2018).

Long non-coding RNA (lncRNA, > 200 nt) is known to be involved in gene expression regulation, genomic imprinting, transcription activation and interference, and intracellular nuclear transport (Chen et al. 2018; Wu et al. 2016). LncRNA dysregulations are closely related to various human disease (Han 2020). LncRNA MAP kinase-activated protein kinase 5 antisense gene protein 1 (MAPKAPK5-AS1) is a newly discovered lncRNA, which has been reported to play important roles in the development of glioma (Luan et al. 2019) and lung cancer (Zhang et al. 2019). In our preliminary experiment, we found that MAPKAPK5-AS1 was dysregulated in LPS treated myocardium. Herein, we are interested to investigate the role of MAPKAPK5-AS1 LPS-induced inflammatory damage in the myocardium.

MicroRNAs (miRNAs) are a type of endogenous non-coding single-stranded RNA consisting of about 21 to 25 nucleotides (Ge et al. 2018). They are mainly paired with the non-transcribed region of the 3′ end of the target mRNA to cleave or inhibit target mRNA translation, thereby affecting protein expression (Mirna et al. 2019). During sepsis, some miRNAs expressions are dysregulated, such as miR-155 (Wang 2016), miR-135a (Zheng et al. 2017), and miR-125b (Ma et al. 2016). Accumulative evidence suggested that miRNAs can regulate the inflammatory response (Ektesabi et al. 2019; Liu et al. 2017). However, due to the complexity of the occurrence and development of sepsis, more experiments are still needed to confirm the reliability and accuracy of these miRNAs as SIMD biomarkers. In our study, we found that MAPKAPK5-AS1 has common binding sites with miR‐124-3p by bioinformatics ananlysis. We also found that miR-124-3p expression was dysregulated in LPS induced myocardium. Therefore, we will focus on their associations and combined role in LPS-induced inflammatory damage in myocardium.

E2F3 is a group of genes that can encode transcriptional regulators and plays an important role in the transformation process (Weintraub et al. 1992; Nevins 1992). E2F3 is an important positive regulator of the cell cycle, which is an important regulatory factor in the G1 to S phase of the cell cycle (Sundarraj and Kannan 2017). E2F3 is closely related to cell metabolism and inflammation. For example, Y. Liao reported that Rb-independent E2F3 promotes cell proliferation and alters the expression of genes involved in metabolism and inflammations in 2017 (Liao and Du 2017). In our study, we found that miR-124-3p could target E2F3 through bioinformatics analysis. We have studied the interactions among MAPKAPK5-AS1, miR-124-3p and E2F3 in LPS-induced inflammatory damage in myocardium. Our result may provide an effective method for the therapeutic directions of myocardial dysfunction.

## Methods

### Animals

Male SD rats (weighing 250–300 g) were obtained from China-Japan Union Hospital of Jilin University and kept at 24 °C with humidity in a half-day and half-dark environment. The rats had the freedom to eat or drink. The experiment protocol was approved by Animal Usage Board of China-Japan Union Hospital of Jilin University. The rats were classified as: (i) vehicle (n = 5); (ii) LPS administration (5 mg/kg) (n = 5); (iii) LPS administration (10 mg/kg) (n = 5) (iv) LPS (10 mg/kg) plus si-MAPKAPK5-AS1 treatment (n = 5). PBS was employed as a carrier buffer. Intraperitoneal injection of 5 or 10 mg/kg LPS (Sigma, Missouri, USA) induced endotoxemia. Rats were injected with lentiviral vectors through the tail vein, and the lentiviral vectors each carried the following plasmids: si-NC, si-MAPKAPK5-AS1. Three days before LPS injection, lentiviral vector injection was performed through the above tail vein (19 × 10^7^ TU per rat). Twenty-four hours after endotoxemia, we treated the rats with 100 mg/kg of ketamine and 10 mg/kg of wood diazine. The hearts were obtained.

### Immunohistochemical analysis of myocardial

CD68 and TNF-α were immunohistochemically stained. The heart tissues were fixed, embedded in paraffin, dewaxed and rehydrated. After blocking, tissues were treated with anti-rat CD68 or TNF-a (Abcam, UK) at 4 °C for a night. The tissues were treated with Chromogen (DakoCytomation, Denmark) and stained with hematoxylin (Sigma, USA). For morphology, tissues were observed by a microscope (Zeiss, Germany) × 400.

### TUNEL

Apoptotic cardiomyocyte and myocardium were detected using a TUNEL kit (Zhongshan Bio., China). Briefly, sections (5 μm) were collected and prepared, deparaffinized and hydrated, and incubated in proteinase K working solution for 30 min at 37 °C. Following washing with PBS twice, they were blocked for 10 min using 3% H_2_O_2_ at room temperature. Subsequently, the sections were incubated in 50 µL terminal deoxynucleotide transferase (TdT) buffer (45 µL Equilibration buffer + 1 µL FITC-12-dUTP + 4 µL TdT Enzyme) for 1 h at 37 °C. After being washed in PBS 3 times, nuclei were stained with DAPI, and TUNEL positive cells were observed and photographed using a fluorescence-capable microscope (Leica DMI4000B, Wetzlar, Germany).

### Cell culture and transfection

Rat cardiomyocyte (H9C2) cells were provided by ScienCell 6200, USA. H9C2 cells were incubated in a 37 °C incubator (with 5% CO_2_ and 95% air) in 10% fetal calf serum (FCS; Invitrogen, USA) and Dulbecco’s modified Eagle’s medium (DMEM; Sigma, USA). miR-124-3p mimic, miR-124-3p mimic, pcDNA3.1-lncRNA MAPKAPK5-AS1 (pc-MAPKAPK5-AS1), si-lncRNA MAPKAPK5-AS1 (si-MAPKAPK5-AS1) and control (Mibo NC, pcDNA3.1-vector, si-NC was synthesized by RiboBio, China. H9C2 cells was plated for 1 day. HiPerFect transfections were employed according to the manufacturer's instructions (QIAGEN, Germany) Cell transfection. Further studies were performed 48 h after transfection.

### Luciferase assays

Sequences of MAPKAPK5-AS1 or E2F3 containing a putative miR-124-3p binding site were amplified by RT-PCR. The sequence was cloned to the pmirGLO luciferase vector (Promega, USA). GeneArtTM (Thermo Fisher Scientific) was employed for miR-1214-3p mutant (MUT) in MAPKAPK5-AS1 or E2F3 sequences. Then, in the presence of miR-124-3p mimics or miR-NC, H9C2 cells had transfection with wild-type (WT) or MUT vector. The luciferase activities were detected by luciferase assays (Promega) and normalized to Renilla.

### Pull-down assay

H9C2 cells had transfections to biotin-labeled WT-miR-124-3p WT or MUT-bio-miR-124-3p. After 2 days, H9C2 cells were washed and lysed and incubated with magnetic bead (S3762; Millipore) and BSA (Sigma). The bead had incubation at 4 °C for 3 h and washed. Finally, Trizol was employed to purify the bound RNA and determine its content.

### Apoptotic cells

Flow cytometry was employed to analyze apoptotic H9C2 cells. Briefly, H9C2 cells (1 × 10^5^) were respectively treated with 5 μL Annexin V and propidium iodide (PI) together for 15 min at room temperature in the dark. After 48 h, the H9C2 cells were collected and washed with cold PBS twice. Cell apoptosis was detected with Annexin V-FITC/PI cell apoptosis kit (130-092-052, Miltenti Biotech, Waltham, MA) in Guava easyCyte Benchtop Flow Cytometer (BR168323; Luminex, Austin, TX). The apoptosis rate was analysed using a Becton–Dickinson FACSC alibur Flow Cytometer (Beckman Coulter, Brea, CA).

### RT-qPCR

RNA was extracted by TRIzol (Takara, Japan), and had reverse transcription to cDNA. RT-PCR was performed by ABI Prism 7700 Sequence Detection System (PE Applied Biosystems, California, USA) using the following reaction conditions. 40 cycles of 95 °C for 30 s, 62 °C for 45 s and 72 °C for 90 s; 72 °C for 10 min. GAPDH was internal control for LncRNA MAPKAPK5-AS1, and U6 was internal control for miR-124-3p. The expressions were calculated by 2^−ΔΔCT^. Primers utilized in this research were:

miR-124-3p: forward 5′-ACA GGC TAA GGC TCC CAGTGA A-3′, reverse 5′-CGC AGG GTC CGA GGTATT C-3′;
MAPKAPK5-AS1: Forward AAGCCCGAGTCTGATGCTAA and reverse CTGCACACCTCTCCTCTGGGGA;
TNF-α: forward 5′-AGC ACA GAA AGC ATG ATC CG-3′, reverse 5′-CTG ATG AGA GGG AGG CCATT-3′;
IL-1β: forward 5′-TGC AGA GTT CCC TTG AAT C-3′, reverse 5′-GTC CTG CCT AAT GTC CCC TTG AAT C-3′;
IL-6: forward 5′-GAG GAT ACC ACT CCC AAC AGACC-3′, Reverse 5′-AAG TGC ATC ATC GTT GTT CATACA-3′;
GAPDH: forward 5′-TGT TCG TCATGG GTG TGA AC-3′, reverse 5′-ATG GCATGG ACT GTG GTC AT-3′;
U6: forward 5′-CTC GCT TCG GCA GCA CA-3′, reverse 5′-AAC GCT TCA CGA ATT TGC GT-3′.
MMP2: forward 5′-GTCAAGTATGGTTGGGCAGTT-3′, reverse 5′-GCC CAAG ATGCC CTTC AGT-3′;
MMP9: forward 5′-GAAGTCTCAGAAGGTGGAT-3′, reverse 5′-GAAATAGGCTTTGTCTTGGTA-3′.

### Western blotting

RIPA buffer (Roche, Germany) was utilized for the protein extraction of H9C2 cells. Protein was loaded and separated by 10% SDS-PAGE and transferred to a PVDF membrane (Millipore, USA). The protein was blocked by 5% skim milk for 2 h, and treated with anti-TNF-a (1:1000, Abcam, UK), anti-GAPDH (1:10,000, Abcam), anti-IL-1b (1:1000, Abcam), anti-Caspase3 (1:1000, CST), anti-PARP (1:1000, Abcam), anti-MCP-1 (1:1000, Abcam), anti-E2F3 (1:1000, Abcam, UK), and anti-IL-6 (1:1000, Abcam, UK) at 4 °C for 12 h. After washing, the membrane was treated with IgG-HRP (ab6802, Abcam, 1:2000, UK) and incubated at room temperature for 2 h. Visualize signals with enhanced chemiluminescence reagent (ECL, Germany).

### Cell counting Kit-8 assay

The cells were seeded at a density of 5000 cells per well in a 96-well plate (Beyotime). After the quantitative treatment, a CCK-8 solution (Bioswamp, Wuhan, China) was added to the culture medium according to the instructions, and the plate was incubated at 37 °C in 5% CO_2_ for 1 h in the dark. Quantify absorbance at 450 nm using a microplate reader (Bio-Rad, Sunnyvale, CA).

### Data methods

All measurements were performed in triplicate. All the results were expressed as mean ± standard deviations. Student's t-test was used for comparison of the two, one-way ANOVA was used for single factor comparison of multiple groups. P < 0.05 was considered statistically significant.

## Result

### MAPKAPK5-AS1 is upregulated, while miR-124-3p is downregulated in myocardial injury induced by LPS

**Fig. 1:**
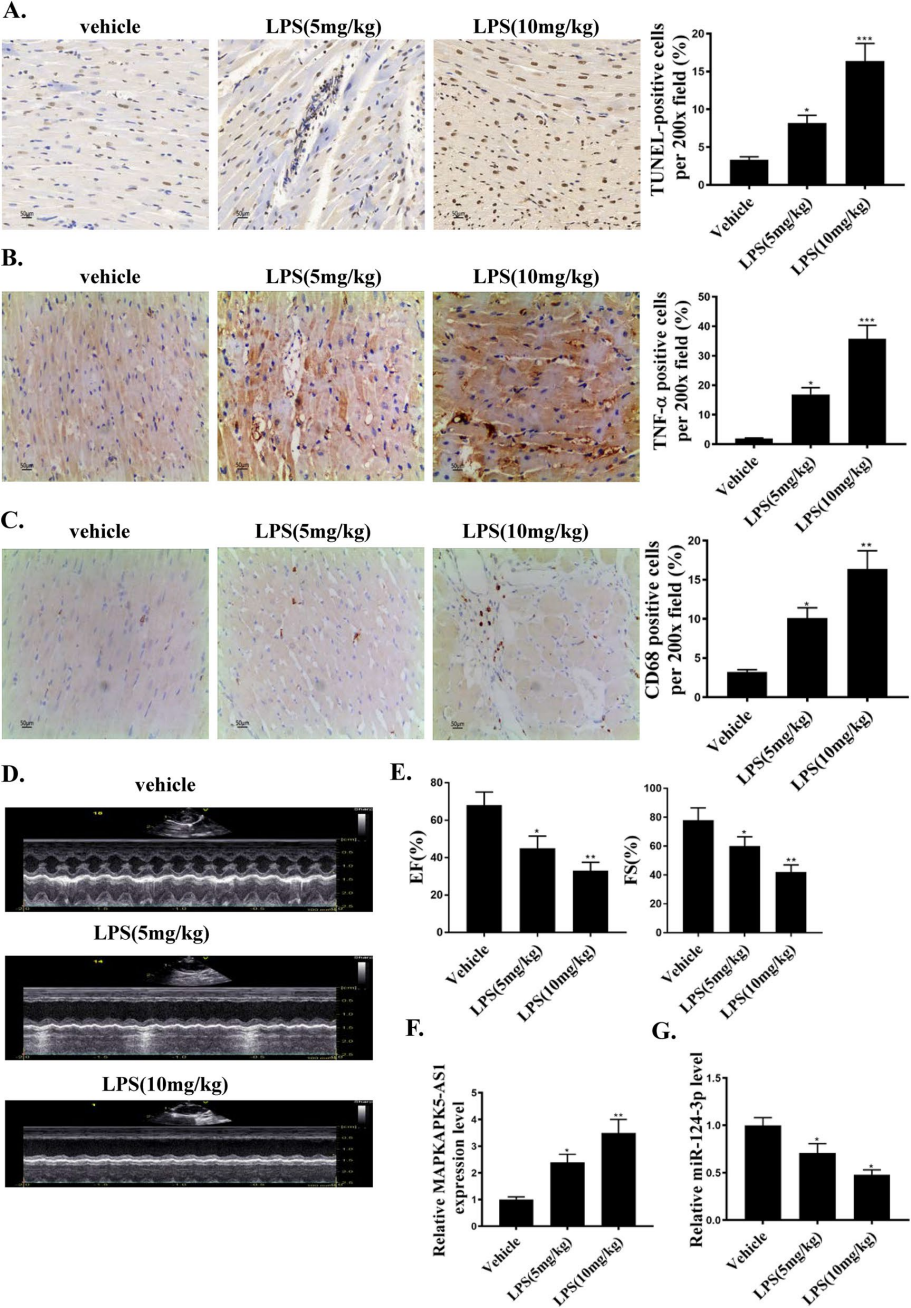
LncRNA MAPKAPK5-AS1 is upregulated, while miR-124-3p is down-regulated in myocardial injury induced by LPS. **A** TUNEL staining for cell apoptosis (gray) in the groups of Vehicle, LPS (5 mg/mg), and LPS (10 mg/kg), scale bar: 50 μm. **B** IHC for TNF-α positive cells (gray) per × 200 field in groups of vehicle, LPS (5 mg/mg), and LPS (10 mg/kg), scale bar: 50 μm. **C** IHC for CD68 positive cells (gray) per 200 × field in the groups of Vehicle, LPS (5 mg/mg), and LPS (10 mg/kg). **D** and **E** LPS treatment reduced the cardiac function in a dose-dependent manner. **F** Relative MAPKAPK5-AS1 expression in heart tissue in the groups of Vehicle, LPS (5 mg/mg), and LPS (10 mg/kg). **G** MiR-124-3p expression in heart tissue in the groups of Vehicle, LPS (5 mg/mg), and LPS (10 mg/kg). Each column represents the mean ± SEM. Statistical differences were evaluated with One-way ANOVA followed by Tukey’s post-hoc test. *P < 0.05, **P < 0.01, ***P < 0.001, n = 5

To evaluate the expression of MAPKAPK5-AS1 and miR-124-3p in myocardial injury induced by LPS, we conducted TUNEL, CD68, and TNFa immunochemical staining, as well as in vivo RT-PCR. Twenty-four hours after LPS treatment, the number of TUNEL-positive cells, CD68 and TNFa-positive cells was increased in a dose-dependent manner (Fig. 1A–C). Moreover, LPS treatment was found to reduce the cardiac function in a dose-dependent manner. In addition, As shown in Fig. 1D and E, LPS treatment reduced the cardiac function in a dose-dependent manner. Furthermore, the expression of MAPKAPK5-AS1 increased while the expression of miR-124-3p decreased (Fig. 1F, G). Overall, we found that MAPKAPK5-AS1 was upregulated and miR-124-3p was downregulated in LPS-related myocardial injury.

### Down-regulation of MAPKAPK5-AS1 attenuated H9C2 cell apoptosis and inflammation response induced by LPS

**Fig. 2:**
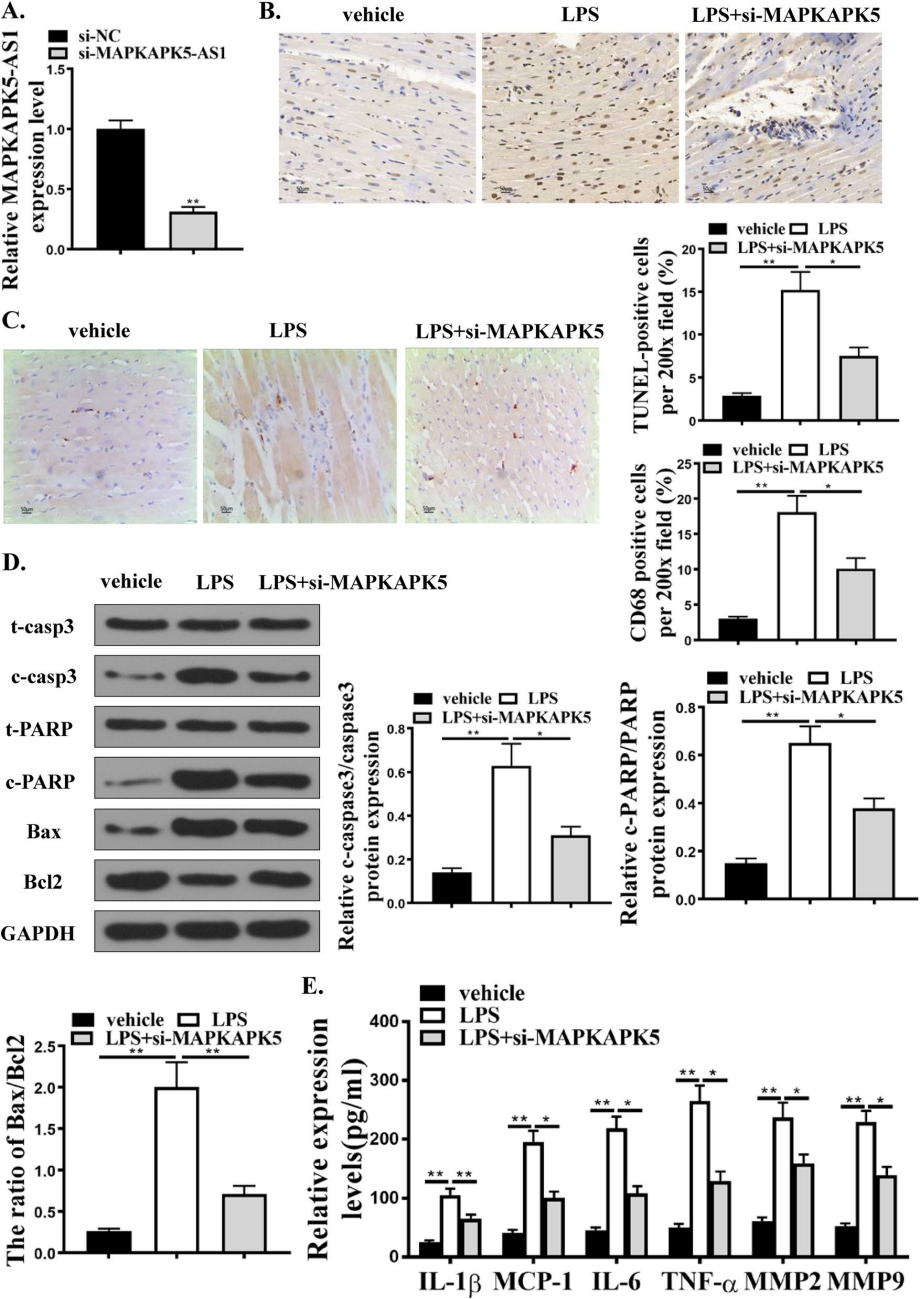
Down-regulation of MAPKAPK5-AS1 attenuates myocardial apoptosis and inflammation induced by LPS. **A** MAPKAPK5-AS1 expression in cardiac tissue. **B** TUNEL staining for cell apoptosis analysis in cardiac tissues. **C** IHC for CD68 positive cells per × 200 field in cardiac tissues. **D** Western blot analysis for the expression of t-casp3, c-casp3, t-PARP, e-PARP, Bax, Bcl2 and GAPDH in cardiac tissues. E. Relative expression levels of IL-1β, MCP-1, IL-6, TNF-α, MMP2, and MMP9 in cardiac tissues. Each column represents the mean ± SEM. Statistical differences were evaluated with One-way ANOVA followed by Tukey’s post-hoc test. *P < 0.05, **P < 0.01, n = 5

In order to explore the role of MAPKAPK5-AS1 in LPS-induced inflammatory damage, rats were pre-injected with a lentiviral vector with si-MAPKAPK5-AS1 3 days before LPS injection. The expression of MAPKAPK5-AS1 in cardiac tissue is shown in Fig. 2A. Compared with the LPS (10 mg/kg) group, si-MAPKAPK5-AS1 reduced TUNEL-, CD68, and TNFa positive cells (Fig. 2B, C). Western blot analysis of caspases3, PARP, and Bax/Bcl2 showed that si-MAPKAPK5-AS1 reduced myocardial cell apoptosis (Fig. 2D). LPS-induced inflammatory cytokines are attenuated by si-MAPKAPK5-AS1 in cardiac tissue (Fig. 2E). It was confirmed that MAPKAPK5-AS1 knockdown attenuated H9C2 cells apoptosis and inflammation response induced by LPS.

### Down-regulation of MAPKAPK5-AS1 reduced the LPS-induced H9C2 cell apoptosis and inflammation response

**Fig. 3:**
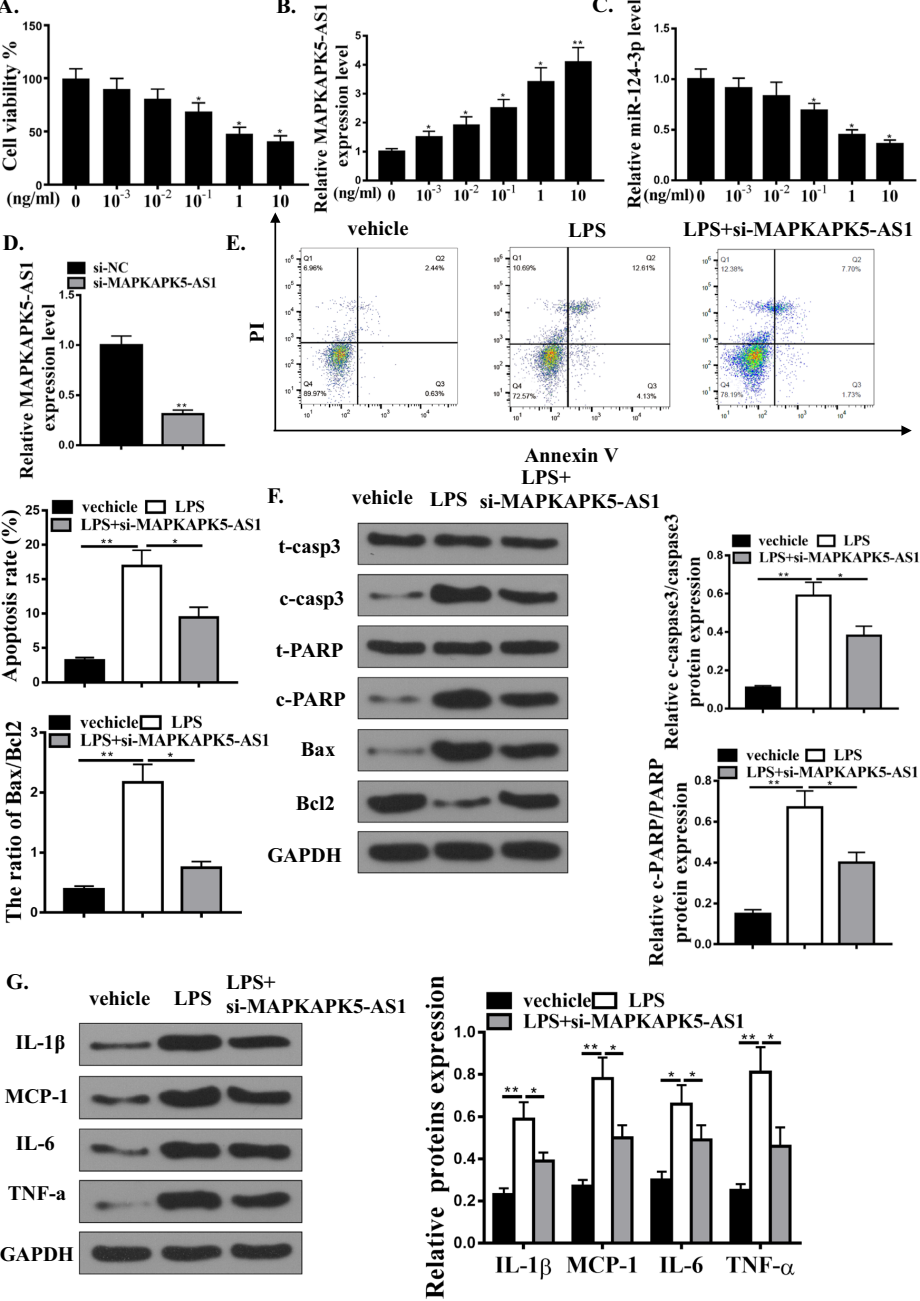
Down-regulation of MAPKAPK5-AS1 reduces LPS-induced rat cardiomyocyte apoptosis and inflammation. **A** Cell viability of H9C2 cells was detected after treated with various concentrations of LPS (0, 0.001, 0.01, 0.1, 1, and 10 ng/mL). **B** The expression of MAPKAPK5-AS1 in H9C2 cells treated by various concentrations of LPS (0, 0.001, 0.01, 0.1, 1, and 10 ng/mL). **C** The expression of miR-124-3p in H9C2 cells treated with various concentrations of LPS (0, 0.001, 0.01, 0.1, 1, and 10 ng/mL). **D** MAPKAPK5-AS1 expression in H9C2 cells transfected by si-NC or si-MAPKAPK5-AS1. **E** Flow cytometry analysis of apoptosis in cells treated with vehicle, LPS, LPS + si-MAPKAPK5-AS1. **F** Western blot for protein expressions of t-casp3, c-casp3, t-PARP, e-PARP, Bax, Bcl2, and GAPDH in H9C2 cells treated with vehicle, LPS, or LPS + si-MAPKAPK5-AS1. **G** Western blot for protein expression levels of IL-1β, MCP-1, IL-6, TNF-α, MMP2, and MMP9 in H9C2 cells treated with vehicle, LPS. or LPS + si-MAPKAPK5-AS1. Each column represents the mean ± SEM. Statistical differences were evaluated with One-way ANOVA followed by Tukey’s post-hoc test. *P < 0.05, **P < 0.01, n = 3

In vitro, H9C2 cells treated with different doses of LPS (0, 10/-3, 10/-2, 10/-1, 1, 10 μg/ml) lasted 24 h, and cell viability was examined using CCK8 (Fig. 3A). As shown in Fig. 3B and C, the expression of MAPKAPK5-AS1 increased in a dose-dependent manner while miR-124-3p decreased. We then tested the role of MAPKAPK5-AS1 in vitro and detected the expression of MAPKAPK5-AS1 by qRT-PCR (Fig. 3D). The expression of MAPKAPK5-AS1 was inhibited by si-MAPKAPK5-AS1. Flow cytometry analysis and Western blot analysis were employed to examine LPS-related apoptosis. H9C2 cells treated with 1 μg/ml LPS for 24 h increased apoptosis, while si-MAPKAPK5-AS1 attenuated LPS-related apoptosis (Figs. 3E and F). Western blot analysis of inflammatory cytokine protein expressions showed that si-MAPKAPK5-AS1 attenuated LPS-related inflammatory response (Fig. 3G). Therefore, MAPKAPK5-AS1 knockdown reduced the LPS-induced H9C2 cell apoptosis and inflammation response.

### miR-124-3p was a target of MAPKAPK5-AS1

**Fig. 4:**
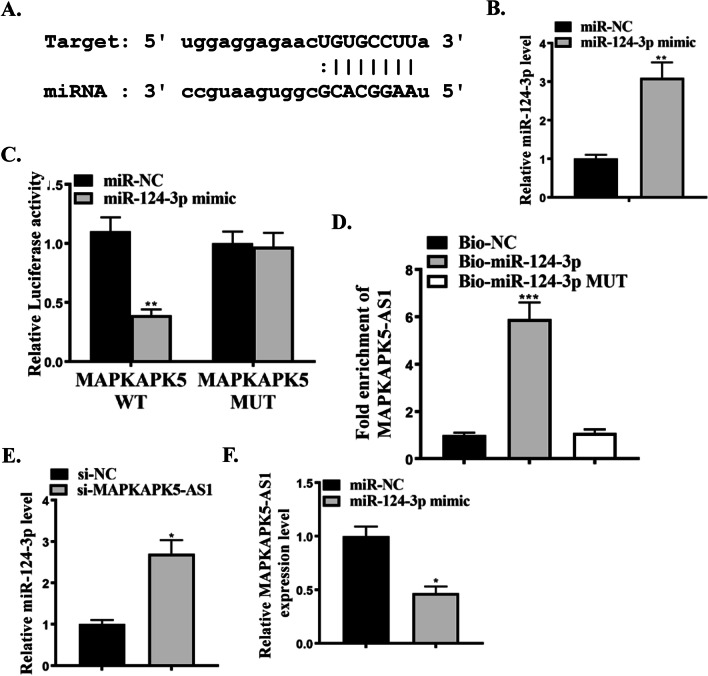
miR-124-3p is one of the targets of MAPKAPK5-AS1. **A** Bioinformatics revealed the common binding sites between MAPKAPK5-AS1 and miR-124-3p. **B** Expression of miR-124-3p in H9C2 cells transfected by miR-NC or miR-124-3p mimic. **C** Relative luciferase activity for cells transfected with miR-NC, miR-124-3p mimic with MAPKAPK5-AS1 WT or MAPKAPK5-AS1 MUT. **D** RNA pull-down for the fold enrichment of MAPKAPK5-AS1 in cells transfected with Bio-NC, Bio-miR-124-3p or Bio-miR-124-3p MUT. **E** Expression of miR-124-3p in H9C2 cells transfected by si-NC, or si-MAPKAPK5-AS1. **F** MAPKAPK5-AS1 expression in H9C2 cells transfected by miR-NC or miR-124-3p mimic. Each column represents the mean ± SEM. Statistical differences were evaluated with Student’s t-test or One-way ANOVA followed by Tukey’s post-hoc test. *P < 0.05, **P < 0.01, ***P < 0.001, n = 3

Here, the relationship between miR-124-3p and MAPKAPK5-AS1 is predicted in Starbase (http://starbase.sysu.edu.cn). MiR-124-3p-specific binding sites were found in the 3'UTR of MAPKAPK5-AS1 (Fig. 4A). Subsequently, a dual-luciferase reporter gene assay was performed to determine whether MAPKAPK5-AS1 is a target gene of miR-124-3p, suggesting that co-transfection with miRNA can attenuate MAPKAPK5-AS1-3'UTR Wt luciferase activity (Fig. 4C), while the MAPKAPK5-AS1-3'UTR-Mut mimic did not change in response to co-transfection of miR-124-3p mimic in H9C2 cells. The same results were obtained by RNA pull-down assay (Fig. 4D). In addition, si-MAPKAPK5-AS1 increased the expression level of miR-124-3p (Fig. 4E), and miR-124-3p mimicked the reduction of MAPKAPK5-AS1 expression level in H9C2 cells (Fig. 4F). These data indicated that miR-124-3p was a target of MAPKAPK5-AS1.

### miR-124-3p mediated the effects of MAPKAPK5-AS1 on LPS-induced H9C2 cell apoptosis and inflammation response

**Fig. 5:**
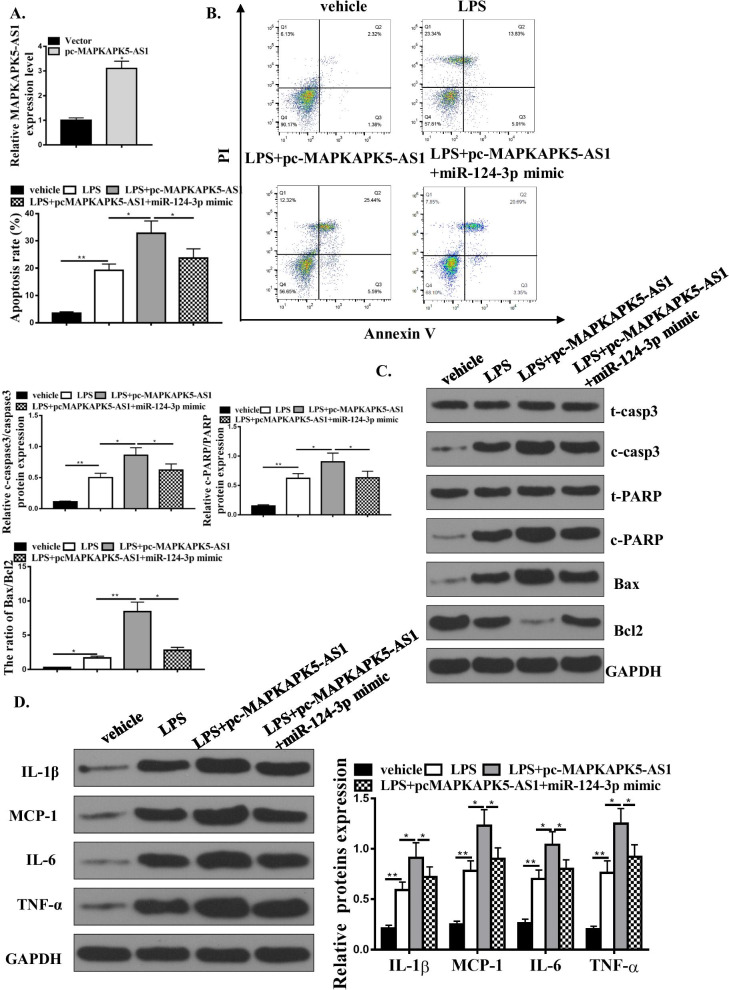
miR-124-3p mediates the effects of MAPKAPK5-AS1 on the apoptosis and inflammatory response of H9C2 cells induced by LPS. **A** MAPKAPK5-AS1 expression in H9C2 cells transfected with vector or pc-MAPKAPK5-AS1. **B** Cell flow cytometry for cells treated by vehicle, LPS + pc-MAPKAPK5-AS1 or LPS + pc-. MAPKAPK5-AS1 + miR-124-3p mimic. **C** Western blot for protein expressions of t-casp3, c-casp3, t-PARP, e-PARP, Bax, Bcl2, and GAPDH in cells treated by vehicle, LPS, LPS + pc-MAPKAPK5-AS1 or LPS + pc-MAPKAPK5-AS1 + miR-124-3p mimic. **D** Western blot for protein expression levels of IL-1β, MCP-1, IL-6, TNF-α, MMP2, and MMP9 in cells treated by vehicle, LPS, LPS + pc-MAPKAPK5-AS1 or LPS + pc-MAPKAPK5-AS1 + miR-124-3p mimic. Each column represents the mean ± SEM. Statistical differences were evaluated with Student’s t-test or One-way ANOVA followed by Tukey’s post-hoc test. *P < 0.05, **P < 0.01, n = 3

Then, we aimed to identify whether miR-124-3p could mediate the effects of MAPKAPK5-AS1 on LPS-related H9C2 cell death and inflammations. Flow cytometry and Western blotting were utilized to confirm LPS-related cell death. 1 ug/ml LPS-treated H9C2 cells for 24 h can increase cell death, while pc-MAPKAPK5-AS1 further enhanced LPS-related cell death, and co-transfection with miR-124-3p mimics can attenuate this effect (Fig. 5B and C). Western blotting of inflammatory cytokine protein expression showed that pc-MAPKAPK5-AS1 enhanced LPS-induced inflammation response and co-transfection with miR-124-3p mimics attenuated this effect (Fig. 5D). Overall, our experiments identified that miR-124-3p mediated the effects of MAPKAPK5-AS1 on LPS-induced H9C2 cell apoptosis and inflammation response.

### miR-124-3p regulates E2F3 expression

**Fig. 6:**
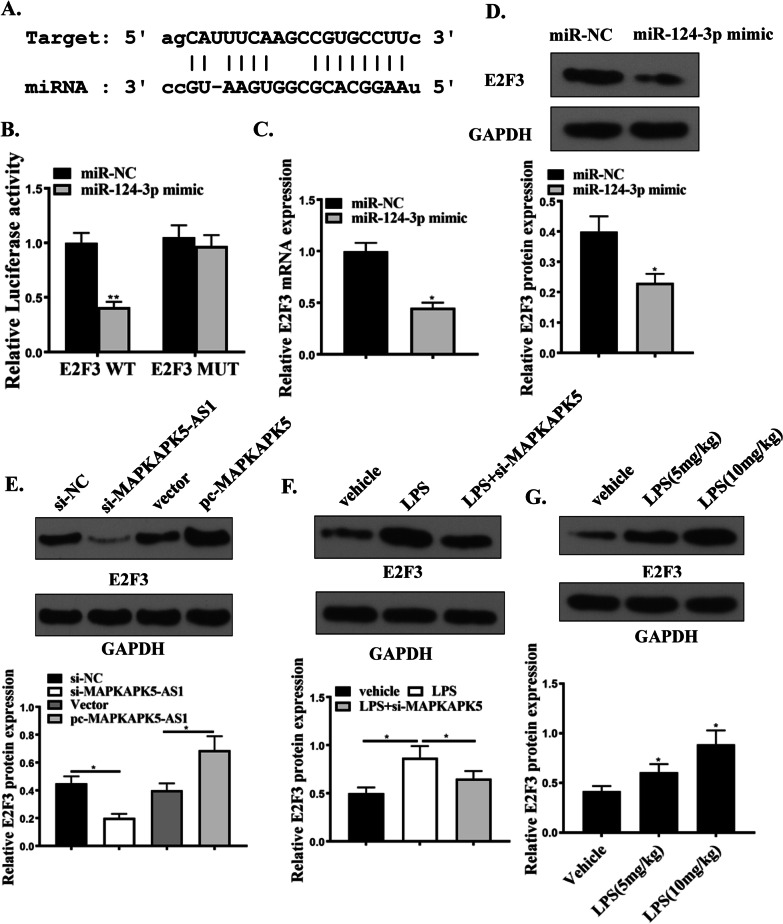
miR-124-3p downregulated E2F3 expressions by direct interactions in H9C2 cells. **A** Bioinformatics for direct interaction between E2F3 and miR-124-3p. **B** Relative luciferase activity for cells transfected by E2F3 WT, E2F3 MUT with miR-NC or miR-124-3p mimic. **C** E2F3 mRNA expression in H9C2 cells transfected by miR-NC or miR-124-3p mimic. **D** E2F3 protein expression in H9C2 cells transfected by miR-NC or miR-124-3p mimic. **E** E2F3 protein expression in H9C2 cellstransfected by si-NC, si-MAPKAPK5-AS1, Vector, or pc-MAPKAPK5-AS1. **F** E2F3 protein expression in cardiac tissue injected by vehicle, LPS, or LPS + si-MAPKAPK5-AS1. **G** E2F3 protein expression in cardiac tissue treated by vehicle, LPS (5 mg/kg), or LPS (10 mg/kg). Each column represents the mean ± SEM. Statistical differences were evaluated with Student’s t-test or One-way ANOVA followed by Tukey’s post-hoc test. *P < 0.05, **P < 0.01, n = 3

**Fig. 7:**
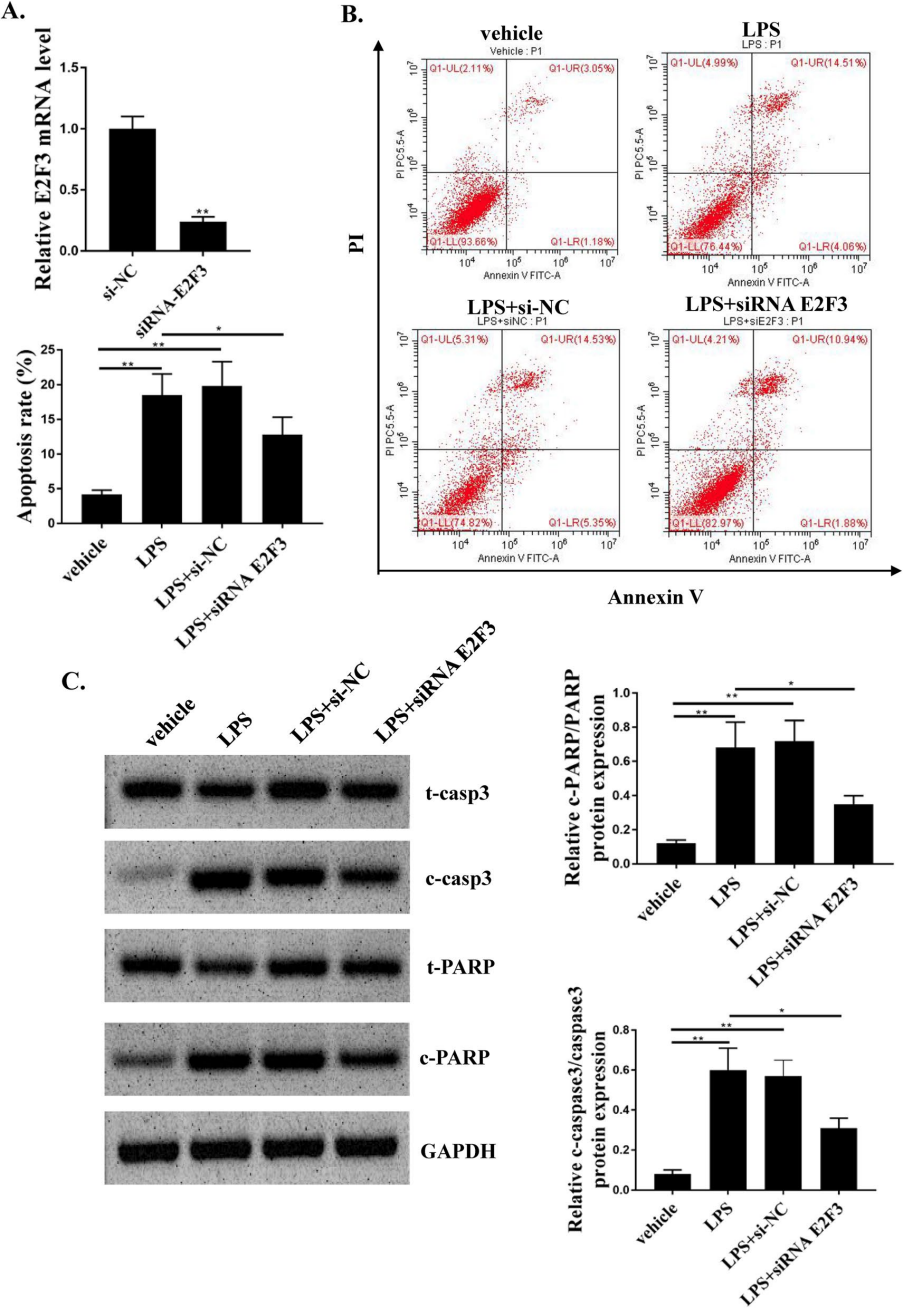
E2F3 involves in the H9C2 cell apoptosis induced by LPS. **A** Knockdown of E2F3 in H9C2 cells. **B** E2F3 knockdown significantly reduced the H9C2 cell apoptosis induced by LPS. **C** E2F3 knockdown significantly reduced the phosphorylation levels of caspases3 and PARP induced by LPS. *P < 0.05, **P < 0.01, n = 3

Here, the relationship between miR-124-3p and E2F3 was predicted in TargetScan, microT and PITA. The specific binding site of miR-124-3p was found in the 3'UTR of E2F3 (Fig. 6A). Subsequently, a dual-luciferase reporter gene assay was performed to determine whether E2F3 is a target gene of miR-124-3p, suggesting that co-transfection with miR-124-3p mimics attenuated E2F3-3'UTR Wt fluorescence Enzyme activity (Fig. 6B), while E2F3-3'UTR-Mut did not change in response to co-transfection of miR-124-3p mimics in H9C2 cells. miR-124-3p mimics E2F3 expressions at mRNA and protein levels (Fig. 6C and D). As shown in Fig. 6E, MAPKAPK5-AS1 regulates the expression of E2F3 in H9C2 cells. In rat heart tissue, the MAPKAPK5-AS1/miR-124-3p axis also regulates E2F3 expression (Fig. 6F). Finally, rats injected with LPS showed high expression of E2F3 (Fig. 6G). Moreover, knockdown of E2F3 significantly reduced the H9C2 cell apoptosis,the phosphorylation levels of caspases3 and PARP induced by LPS (Fig. 7A-C). Our results confirmed that miR-124-3p downregulated E2F3 expression in H9C2 cells.

## Discussion

SIMD is a life-threatening dysfunction caused by a host's dysregulated response to infection, which is a common clinical critical illness and a serious public health problem worldwide (Cheng 2019). However, the underlying pathogenesis of SIMD remains unknown. SIMD can trigger a series of complex and interrelated pathophysiological processes, such as genetic polymorphisms, immune dysfunction, coagulation disorders and tissue damage (Kim et al. 2019), but their related molecular mechanisms need to be further studied (Jain et al. 2018). In this study, we found that MAPKAPK5-AS1 was upregulated, and miR-124-3p was downregulated in myocardial injuries induced by LPS, and MAPKAPK5-AS1 promoted LPS induced inflammatory damage in the myocardium by sponging microRNA-124-3p/E2F3.

It has been reported that lncRNA plays an important role in LPS-induced inflammatory response disorder. For example, lncRNA PVT1 induces an increase in TNF-α, IL-6, and IL-1β release, and promotes inflammation by regulating TNF-α and JNK/NF-κB signaling pathways in sepsis (Huang et al. 2017). Fang et al. find that lncRNA H19 reduces the expression of miR-874, downregulates the secretion of inflammatory factors, and restores LPS-induced inflammatory response disorder and myocardial dysfunction in sepsis mice(Fang et al. 2018). NEAT1 in circulating blood is associated with increased disease risk, ascending severity of the disease, poor prognosis, and rising expression of inflammatory factors in sepsis patients (Huang et al. 2018). MAPKAPK5-AS1 is located on chromosome 12q24.12 and has been demonstrated to act as an oncogenic molecule in many kind of cancers, such as hepatocellular carcinoma, thyroid cancer and colorectal cancer et.al (Yang et al. 2020; Zhou et al. 2020). In our study, we investigated the role of MAPKAPK5-AS1 in inflammatory damage induced by LPS, and we found that MAPKAPK5-AS1 knockdown could reduce the increased number of TUNEL-, CD68 and TNFa positive cells and H9C2 cell apoptosis induced by LPS. Moreover, MAPKAPK5-AS1 knockdown attenuated LPS-induced inflammatory response. These results suggest that MAPKAPK5-AS1 knockdown attenuated H9C2 cell apoptosis and inflammation response in myocardial injuries induced by LPS.

Previous studies have pointed out that lncRNA could act as the sponge of miRNA and regulate the activities of HCMs (Piccoli et al. 2015; Kataoka and Wang 2014) and SIMD (Chen et al. 2018; Fang et al. 2018). Based on the bioinformatics results from Starbase, dual-luciferase reporter gene assay, and RNA pull-down assay, we found that miR-124-3p was a target of MAPKAPK5-AS1. Besides, we also found LPS induced cell death, MAPKAPK5-AS1 overexpression further enhanced cell death, but co-transfection with miR-124-3p mimics can attenuate this effect. Moreover, MAPKAPK5-AS1 overexpression enhanced LPS-induced inflammation reponse, but co-transfection with miR-124-3p mimics attenuated this effect. These results demonstrated for the first time that miR-124-3p could mediate the effects of MAPKAPK5-AS1 on LPS-induced H9C2 cell apoptosis and inflammation response.

It has been reported that E2F3 plays an important role in the development of HCMs (King et al. 2008), and miRNAs are found to be involved in regulate cardiomyocytes cell cycle re-entry (miR-128) (Huang, et al. 2013) or protect cardiomyocytes by inhibiting or targeting E2F3 (miR-210) (Bian et al. 2018). Wang et al. found that miR-124-3p could bind with E2F3 to regulate the properties of Osteosarcoma cells (Wang et al. 2019). In this study, using bioinformations analysis and dual-luciferase reporter gene assay, we also found that E2F3 was a target gene of miR-124-3p. It is consistent with the previous study conducted by Wang et al. (Wang et al. 2019). Moreover, MAPKAPK5-AS1 was found to be able to regulate the expression of E2F3 in H9C2 cells and rat heart tissue. Taken together, our study firstly proposed that miR-124-3p/ E2F3 axis mediated the effect of MAPKAPK5-AS1 on LPS-induced inflammatory response. Additionally, it’s known that there are a series of miRNAs that have multiple target genes, and may participate in different pathogenic processes by regulating one or more target gene(s). For miR-124-3p, there are also different targets that have been studied. Whether one or more other target genes mediated the role of miR-124-3p in LPS-induced inflammatory response needed to be further investigated. Meanwhile, more details involved in the effects of miR-124-3p on LPS-induced inflammatory response should be well studied in the future.

## Conclusion

In summary, our study revealed a novel regulatory model of MAPKAPK5-AS1/miR-124-3p/E2F3 axis in the progression of H9C2 cell apoptosis and inflammatory response induced by LPS.

## Data Availability

The datasets used analyzed during the current study are available from the corresponding author on reasonable request.
